# Enlargements of the Liver.—VII

**Published:** 1910-02-19

**Authors:** 


### ENLARGEMENTS OF THE LIVER.—VII.

### Pernicious Anæmia.

The liver may be definitely enlarged in pernicious anæmia, and there may also be slight or moderate enlargement of the spleen. At a post-mortem examination the liver often presents a characteristic pale russet-brown appearance. It also has an excess of iron, especially in the outer and middle zones of the lobules, as shown very prettily by the prussian blue reaction with the potassium ferrocyanide and hydrochloric acid test. The chief symptoms are marked asthenia and anæmia and primrose yellow tint of the skin. The diagnosis cannot be made until the blood has been carefully examined. The colour of the fresh drop is almost distinctive. The red corpuscles are much reduced in number, often to about 1,000,000 per cm. Quincke recorded a case in which they fell as low as 147,000 per cm. A large proportion (55 to 90 per cent.) of the red cells are megalocytes. Megaloblasts are often present, though they may be numerous at one time, scanty at another. Poikilocytosis and polychromatophilia are present in decided cases. The hæmoglobin is much diminished, and yet less so than are the red cells, at any rate at some period of the disease; so that, notwithstanding the severity of the anæmia, the colour index is not diminished; it is indeed usually higher than normal, lying somewhere between 1 and 1.75. There is leucopænia, and a differential leucocyte count shows a relative lymphocytosis. An occasional basophil leucocyte and one or two myelocytes are to be seen. The treatment is essentially by the use of arsenic.

### Rickets.

The liver as well as the spleen may be slightly or even moderately enlarged in rickets. Nevertheless it needs to be remembered that, owing to the alteration in the shape of the chest caused by the disease, the liver is less well covered by the ribs than it should be. Consequently it may appear to be enlarged when it is not, as the edge of the normal organ can be felt a considerable distance below the costal margin. The enlargement of the liver in rickets may cause no symptoms, and if there is much deformity of the thorax it will be very difficult to say whether the liver is really enlarged or not. The diagnosis of the cause will depend on the coexistence of other signs of rickets.

### Signs of Rickets.

In the early stages of the disease there is general tenderness of the whole body, particularly along the bones, slight pyrexia, restlessness at night, fatness and overweight; unless intercurrent maladies, such as diarrhœa and vomiting, or bronchitis and broncho-pneumonia lead to marasmus, great restlessness and profuse sweating about the head and neck at night. Nodules develop at the junctions of the ribs with the costal cartilages forming the so-called "rickety rosary"; the sternum may be pushed forward, causing the "pigeon breast" chest. A depression extends downwards and outwards from just outside the costo-chondral articulations, and a transverse groove (Harrison's sulcus) extends outwards from the ensiform cartilage into the axilla, with eversion of the ribs below this groove. The head becomes rectangular (caput quadratum) from excess of material for bone formation accumulating at the frontal and parietal eminences; and yet deficiency in the formation of good bone leads to the fontanelles remaining open months longer than they should. The anterior fontanelle should close about the fourteenth month. Occasionally the bones in the parieto-occipital region may be so soft and thinned that they yield to pressure (cranio-tabes). Teething is late, and the teeth are small, badly formed, and irregular in their order of eruption. The lower ends of the radius, ulna, tibia, and fibula are thickened. The natural curves of the bones of the limbs become pathologically exaggerated. The abdomen is usually large and prominent, partly from flatulence, partly from diminution in the size of the thorax, and partly from enlargement of the liver, spleen, and abdominal lymphatic glands.

